# miR-542-3p-Targeted PDE4D Regulates cAMP/PKA Signaling Pathway and Improves Cardiomyocyte Injury

**DOI:** 10.1155/2022/7021200

**Published:** 2022-03-19

**Authors:** Yu Lu, HuaJun Wu, Min Deng, MingDe Huang, HaiHua Pan, PingShao Yang

**Affiliations:** Affiliated Hospital of Jiaxing University, The First Hospital of Jiaxing, Jiaxing 314000, Zhejiang, China

## Abstract

**Objective:**

To investigate the protective effect of miR-542-3p on cardiomyocyte injury and related mechanisms.

**Methods:**

A cardiomyocyte hypoxia/reoxygenation model was established. The expression levels of miR-542-3p and PDE4D were detected using qRT-PCR; the luciferase reporter assay system was used to detect the targeting relationship between miR-542-3p and PDE4D; overexpressing miR-542-3p was transfected into cardiomyocytes, and ROS release was detected by immunofluorescence while cellular apoptosis was detected by TUNEL; and the western blot assay was applied to detect the expression of PDE4D, phosphorylated protein kinase A (p-PKA), and phosphorylated cyclic adenosine monophosphate (cAMP) response element-binding protein (p-CREB).

**Results:**

Compared with the control group, the miR-542-3p expression level was decreased and the PDE4D expression level was increased in the cardiomyocyte hypoxia/reoxygenation model group. The dual-luciferase reporter assay system confirmed that miR-542-3p could target and regulate PDE4D; the transfection with cardiomyocytes using the overexpressing miR-542-3p could downregulate PDE4D expression, attenuate ROS release during cardiomyocyte injury, and reduce cellular apoptosis rate, while upregulating the expression of p-PKA and p-CREB.

**Conclusion:**

The miR-542-3p can negatively regulate PDE4D protein expression and attenuate cardiomyocyte injury through a mechanism related to the activation of the cAMP/PKA signaling pathway.

## 1. Introduction

As stated in the Report on Cardiovascular Health and Diseases in China, the cardiovascular disorder has become one of the major public health issues in China. In recent years, with the development of treatments such as coronary artery intervention or bypass grafting, an increasing number of studies have demonstrated that this method can restore blood reperfusion in the ischemic myocardium. However, the resulting side effect is myocardial ischemia/reperfusion (I/R) injury, which has become one of the important reasons for poor prognosis of patients, and determining the pathogenesis of I/R will play a critical role in improving the survival rate of patients [1].

The cAMP/PKA signaling pathway is one of the classical intracellular signaling transduction pathways, where the signal is first transmitted by G protein-coupled receptors to adenylate cyclase, so as to control the cAMP content and accordingly regulate the activity of PKA. The cAMP phosphorylates specific proteins to p-PKA through PKA, causing cellular effects [2, 3]. The cyclic-AMP response element-binding protein (CREB) is a transcription factor located in the cellular nucleus and an effector of the cAMP signaling pathway, and it is involved in a variety of normal cellular physiological activities such as cellular proliferation, differentiation, and survival [4]. Its activated form is phosphorylated CREB (p-CREB). The catalytic subunit of PKA translocates into the cellular nucleus to phosphorylate CREB, promote its binding with the ancillary transcription factor, and trigger the target gene transcription process, thereby regulating the bioactive response and homeostasis in the cells [5]. Chen et al. [6] have demonstrated that sevoflurane post-treatment may protect the myocardium of cardiac I/R rats by activating the cAMP/PKA signaling pathway to attenuate the inflammatory response and inhibit cardiomyocyte apoptosis. Inserte et al. [7] have demonstrated that elevated cAMP levels in myocardial tissues promote the activation of p-PKA and subsequently produce protective effects on cardiomyocytes through Ca^2+^channels in cardiomyocytes. Dai et al. [8] have revealed that endostatin overexpression may attenuate cardiac hypertrophy through the regulatory mechanism of the cAMP/PKA signaling pathway. Gong et al. [9] have investigated the protective effect and mechanism of morphine on injured myocardium, indicating that morphine may attenuate myocardial ischemia-reperfusion injury in rats through the cAMP/PKA signaling pathway. These abovementioned studies suggest that the cAMP/PKA signaling pathway plays an important role in the protection of cardiomyocytes [10]. Phosphodiesterase 4D (PDE4D), a member of the nucleotide phosphodiesterase superfamily, has a role in regulating intracellular cAMP levels [11, 12]. Studies have confirmed that the application of PDE4D inhibitors can significantly inhibit the cAMP/SIRT1/Akt signaling pathway, which protects the brain and prevent brain injury [13]. Lehnart et al. [14] have shown that the inactivation of the PDE4D gene in rats may cause a series of cardiomyopathies, induce heart failure and arrhythmias, and promote myocardial infarction. In another study [15], a small amount of PDE activation at the sinus node and secondary pacing sites can cause tachycardia, enhance contractile activity, and increase pacing excitability, which promotes Ca^2+^ overload, induces the apoptosis of cardiomyocytes, and serves as one of the main leading causes of heart failure.

MicroRNA (miRNA) is a class of small noncoding RNAs, which is mainly responsible for regulating the expression of related genes by binding to mRNA [16, 17]. Studies have shown that abnormal miRNA expression is closely related to the occurrence and development of various cardiovascular disorders, such as cerebral ischemia and myocardial ischemia/reperfusion injury [18]. In this study, we have established a cardiomyocyte hypoxia/reoxygenation model and selected PDE4D as a target so as to predict the related miRNA with a targeted regulation effect, explore its relationship with the cAMP/PKA signaling pathway against myocardial injury, and provide a theoretical basis for targeted therapy research of myocardial injury.

## 2. Materials and Methods

### 2.1. Materials

#### 2.1.1. Main Reagents and Instruments

Rat cardiomyocytes (H9c2 cells) were purchased from the Cell Bank of the Chinese Academy of Sciences. The reagents and instruments used in this study are Trizol extraction solution (Thermo Fisher), reverse transcription kit (Thermo Fisher), Lipofectamine 3000 transfection reagent (Thermo Fisher), RIPA lysate, one-step TUNEL apoptosis assay kit (green fluorescence) (Shanghai Beyotime Biotechnology), DMEM medium (Hyclone, USA), fetal bovine serum (Gibco, USA), reactive oxygen species assay kit (Beijing Solarbio Life Sciences), PDE4D (Abcam, UK), cAMP (CST, USA), PKA (CST, USA), *β*-actin and HRP-labeled secondary antibody (Shanghai Beyotime Biotechnology), electrophoresis and transfer membrane reagents (Shanghai Beyotime Biotechnology), ECL luminescent solution (Millipore, USA), fluorescence microscope (Thermo Fisher), transfer electrophoresis tank (Bio-Rad, USA), and CO_2_ cell incubator (Thermo Fisher).

#### 2.1.2. Cell Grouping and Model Establishment

H9c2 cells were cultured in DMEM low-sugar medium containing 10% fetal bovine serum at 37 °C with 5% CO_2_. The experiment was divided into three parts: the first part of the experiment was randomly divided into the control group and model group; the second part of the experiment was randomly divided into the model group and miR-542-3p overexpression group; the third part of the experiment was randomly divided into the model group and PDE4D overexpression group. The cardiomyocyte hypoxia/reoxygenation model was established with reference to the literature: cell models were produced using 4 mmol/L Na_2_S_2_O_4_ in the conditions of hypoxia for 30 min and reoxygenation for 1 h [19].

### 2.2. Methods

#### 2.2.1. RT-PCR Assay

RNA was extracted from the cells in each group by using Trizol, and cDNA was synthesized according to the operating instructions of the reverse transcription kit. The RT-PCR assay was performed to detect the expression levels of miR-542-3p and PDE4D, using cDNA as the template. U6 and *β*-actin were used as the internal reference. Primers are listed in Table 1:

#### 2.2.2. Fluorescence Detection of Dual-Luciferase Reporter Gene

The sequence of miR-542-3p binding with PDE4D was predicted using the bioinformatics software, and the PDE4D 3′-UTR sequence and the mutated PDE4D 3′-UTR sequence were designed and synthesized. The two target gene fragments were cloned into the luciferase reporter gene vector, to construct dual-luciferase reporter gene mutant vector and wild-type vector, respectively. When the cell density reached 50%, the firefly luciferase plasmid and renilla luciferase plasmid were cotransfected with the cells. After 24 hours of transfection, luciferase activity was determined using the dual-luciferase assay system.

#### 2.2.3. Cell Transfection

Cells grown in logarithmic phase were inoculated into the 6-well plates at a density of 1 × 10^4^/ml per well; 5 *μ*l of Lipofectamine 3000 and 5 *μ*l of the plasmid to be transfected were added to 250 *μ*l of serum-free DMEM medium, mixed thoroughly, and placed for 15–20 min at room temperature; subsequently, the DMEM medium was aspirated, cells were rinsed once with serum-free DMEM medium, which was replenished with 2 ml of serum-free DMEM medium; then, the mixed solution was dripped into the cells, and the plate was gently shaken to thoroughly mix them, and cells were incubated for 4 h at 37 °C in 5% CO_2_; and the incubation continued after the medium was completely replenished.

#### 2.2.4. Reactive Oxygen Species Assay

Cells in each group were collected. 100 *μ*l of the staining working solution was dropwised to the cell suspension (staining working solution was prepared as follows: dilution with O13 Reactive Oxygen Species Fluorescent Probe 40 times), which was incubated at room temperature for 1 h (avoiding light). Cells were rinsed with PBS three times and centrifuged at 1000 rpm for 5 min for each. After mounting, cells were observed under a fluorescence microscope, and images were acquired.

#### 2.2.5. Cellular Apoptosis Assay

Cells in each group were collected and washed once with 1×PBS. Cells were fixed with 4% paraformaldehyde for 30 min. After 1×PBS wash, cells were incubated with PBS containing 0.3% Triton X-100 for 5 min at room temperature. Subsequently, 50 *μ*l of TUNEL assay solution was added, and cells were incubated for 60 min at 37 °C and protected from light. Cells were washed three times with 1×PBS. Cells were mounted using an antifluorescence quenching solution and then observed under a fluorescence microscope.

#### 2.2.6. Western Blot Assay

Cells in each group were collected and lysed in RIPA lysis buffer containing PMSF for 20 min on ice, then centrifuged at 10000 r/min for 15 min, and the supernatant was collected as the total protein. The aliquots of extracted proteins were subjected to SDS-PAGE electrophoresis and blocked for 2 h using 5% skim milk powder (TBST dilution) after transferring the membrane. After the blocking, they were incubated with primary antibodies, PDE4D (1 : 2,000 dilution), cAMP (1 : 2000 dilution), PKA (1 : 2000 dilution), and GAPDH (1 : 5000 dilution) at 4 °C overnight. On the next day, the membrane was washed three times with TBST, for 10 min each, and incubated with secondary antibodies (1 : 1000 dilution) for 1 h at room temperature. After the incubation with secondary antibodies was completed, the membrane was washed three times with TBST, for 10 min each. Then, the luminescence was performed by ECL, and the results were analyzed by using the ImageJ software.

### 2.3. Statistical Analysis

Data were analyzed using the SPSS 20.0 software, and results in each group were expressed as mean ± SD. The data among groups were compared using the *t*-test, and a *P* < 0.05 level indicated that the difference was statistically significant.

## 3. Results

### 3.1. Expression of miR-542-3p and PDE4D in Cardiomyocyte Injury Models

**Figure 1:**
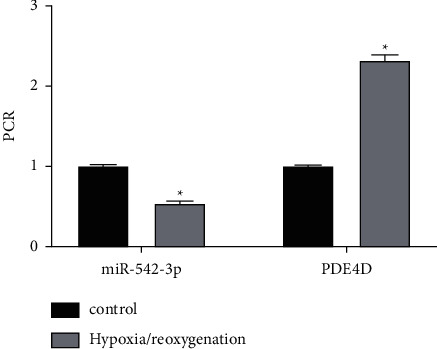
The expression of miR-542-3p and PDE4D in cardiomyocyte injury model. ^*∗*^*p* < 0.05, compared with the control group.

The expressions of miR-542-3p and PDE4D in the control group and model group were detected with the RT-PCR method. As shown in Figure 1, compared with control group, miR-542-3p expression was significantly decreased, whereas PDE4D expression was significantly increased in the model group (*P* < 0.05).

### 3.2. Targeted Relationship between miR-542-3p and PDE4D

**Figure 2:**
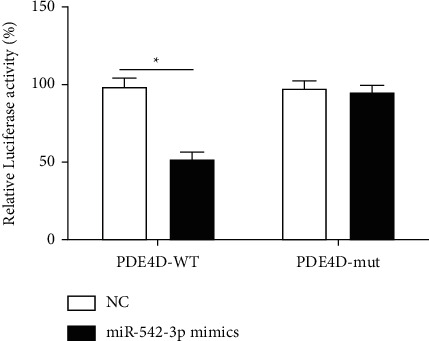
Dual-luciferase report verifies that PDE4D is the target gene of miR-542-3p. ^*∗*^*P* < 0.05, there are statistical differences between the two groups.

To verify that PDE4D is a target gene of miR-542-3p, we performed a dual-luciferase reporter assay. As shown in Figure 2, the luciferase of the wild-type cotransfection group was significantly decreased compared with the negative control group, and the difference was statistically significant (*P* < 0.05); whereas the luciferase signal of the mutant cotransfection group did not decrease significantly, without statistical significance (*P* > 0.05). The abovementioned findings suggested that PDE4D has a direct binding site to miR-542-3p, and activation of miR-542-3p may inhibit the expression of PDE4D.

### 3.3. Effect of miR-542-3p Overexpression on PDE4D Expression in H9c2 Cells with Hypoxia/Reoxygenation Injury

**Figure 3:**
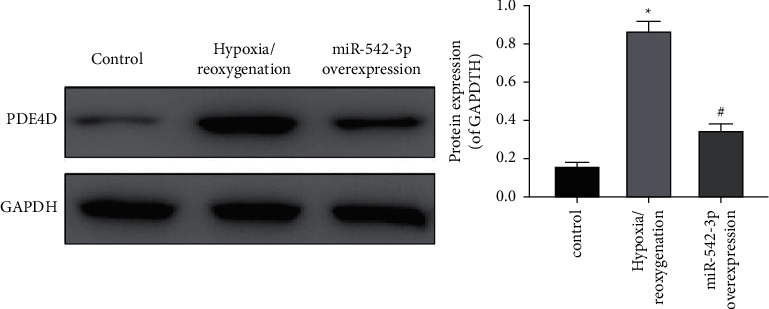
PDE4D expression detected by western blot. ^*∗*^*P* < 0.05, compared with the control group; ^#^*P* < 0.05, compared with the model group.

The overexpressing miR-542-3p vector was constructed and transfected with H9c2 cells to establish a hypoxia/reoxygenation injury model. The PDE4D expression was detected by western blot, and the result is shown in Figure 3. Compared with the hypoxia/reoxygenation injury model group, the PDE4D expression decreased after miR-542-3p was overexpressed.

### 3.4. Effect of miR-542-3p Overexpression on ROS Release in H9c2 Cells with Hypoxia/Reoxygenation Injury

**Figure 4:**
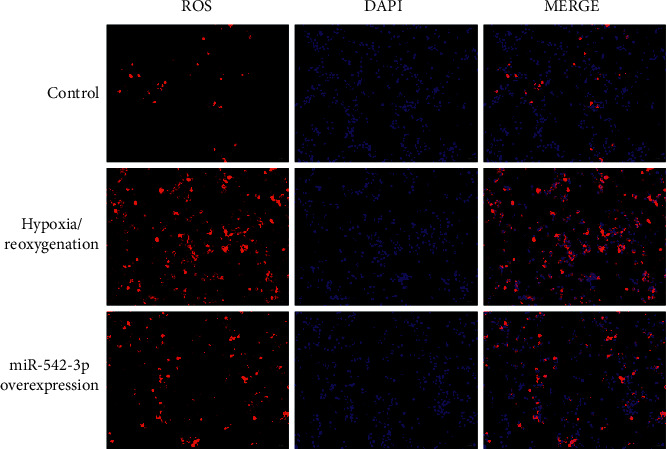
Immunofluorescence detection of ROS.

The results of ROS release by immunofluorescence detection are shown in Figure 4. Compared with the control group, a large amount of ROS was released from H9c2 cells after hypoxia/reoxygenation injury; compared with the model group, the expression of ROS in the miR-542-3p overexpression group was significantly decreased (*P* < 0.05), indicating that the overexpression of miR-542-3p could reduce the release of ROS from myocardial injury cells.

### 3.5. Overexpression of miR-542-3p Reduces the Apoptosis of H9c2 Cells with Hypoxia/Reoxygenation Injury

**Figure 5:**
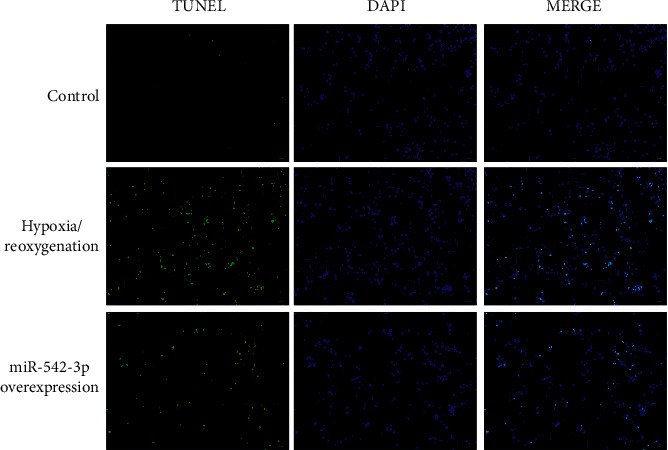
TUNEL detects cell apoptosis.

The apoptosis was observed by TUNEL, as shown in Figure 5. Compared with the control group, the apoptosis rate of H9c2 cells with hypoxia/reoxygenation injury was increased, and there was a statistical difference between the two groups (*P* < 0.05); compared with the model group, the apoptosis rate was reduced in the miR-542-3p overexpression group (*P* < 0.05), indicating that the overexpression of miR-542-3p could reduce the apoptosis rate of myocardial injury cells.

### 3.6. Overexpression of miR-542-3p Regulates the cAMP/PKA Signaling Pathway in Myocardial Injury Cells

**Figure 6:**
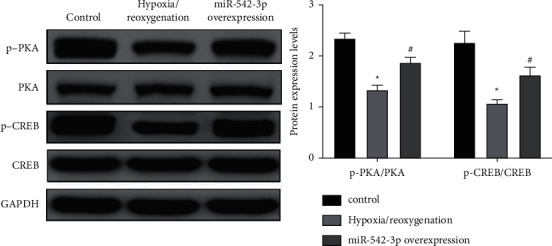
Western blot detects the expression of p-PKA and p-CREB. ^*∗*^*P* < 0.05, compared with the control group; ^#^*P* < 0.05, compared with the model group.

The expressions of phosphorylated protein kinase A (p-PKA) and phosphorylated cyclic adenosine monophosphate (cAMP) response element-binding protein (p-CREB) were detected by the western blot assay, as shown in Figure 6. Compared with the control group, p-CREB and p-PKA expressions were decreased in the hypoxia/reoxygenation injury model group, with a statistical difference between the two groups (*P* < 0.05); compared with the model group, the overexpression of miR-542-3p activated the cAMP/PKA signaling pathway, and the expressions of p-CREB and p-PKA were increased, with a statistical difference between the two groups (*P* < 0.05).

## 4. Discussion

Cardiovascular disorder is one of the diseases with very high morbidity and fatality rate worldwide. In clinical treatment, the myocardium is susceptible to reinjury after the ischemic myocardium restores blood flow, and this process is called ischemia-reperfusion injury [20]. In this study, we establish a hypoxia/reoxygenation model using rat cardiomyocyte H9c2 cells and perform related experiments. The results show that cardiomyocyte injury results in the decreased expression of miR-542-3p and increased expression of PDE4D. Previous studies have shown that miR-542-3p expression levels are abnormally decreased in ischemic stroke [21] and elevated miR-542-3p levels can attenuate the cerebral ischemia-reperfusion injury in mice [22]. In the present study, the results of the dual-luciferase reporter assay system suggest that miR-542-3p could negatively regulate the expression of PDE4D, implying that PDE4D plays an important role in cardiomyocyte injury.

Previous studies have shown that PDE4D can specifically hydrolyze cAMP and is of great significance in the cAMP/PKA signaling pathway [23]. cAMP is an important substance involved in cellular biological functions and is a “second messenger” for information transmission. *In vivo*, cAMP can increase the survival of cardiomyocytes and enhance cardiomyocyte resistance to injury. Studies have demonstrated that cAMP can phosphorylate target proteins through activation of the downstream PKA signaling pathway, which accordingly produces corresponding biological functions [24], and modulation of the cAMP/PKA signaling pathway can attenuate myocardial ischemia-reperfusion injury in rats [25]. To confirm that miR-542-3p can negatively regulate PDE4D expression and ameliorate cardiomyocyte injury, we upregulated the expression level of miR-542-3p using the transfection technique and then analyzed the ROS content, cellular apoptosis, and protein expression levels of PDE4D, cAMP, and PKA, respectively. Overexpression of miR-542-3p is found to attenuate ROS release, reduce cellular apoptosis, decrease PDE4D protein expression, and promote cAMP and PKA protein expression in myocardial injury cells. The abovementioned findings suggest that the elevated miR-542-3p level in cardiomyocytes can negatively regulate the expression of PDE4D protein, thus enabling the activation of the intracellular cAMP/PKA signaling pathway and improving cardiomyocyte injury.

## 5. Conclusion

In summary, the elevated miR-542-3p expression can negatively regulate PDE4D protein expression, reduce the apoptosis of cardiomyocytes, promote the expression of cAMP and PKA in myocardial tissues, protect cardiomyocytes, and attenuate ischemia-reperfusion injury, which provides a theoretical basis and a novel therapeutic target for the treatment of ischemia-reperfusion injury.

## Figures and Tables

**Table 1:** Primer sequence table.

miR-542-3p (F)	5′-UCCAGUCGUGUAAUAGCUCAA-3′
miR-542-3p (R)	5′-CACUUAACACAGAACUGGAUU-3′
U6 (F)	5′-CCTTGAGCATGTCAGAG-3′
U6 (R)	5′-GCACCGTGACTCACCTT-3′
*β*-actin (F)	5′-CAAAGACCTGATCGGACAC-3′
*β*-actin (R)	5′-AGTCCTTGCCGTCACCAGGAG-3′

## Data Availability

I confirm that the data availability of this study is in full compliance with relevant regulations. The original data can be obtained from the corresponding author.
